# Together, at a distance: experiences with a novel technology for social contact among older people and their relatives in Norway during the COVID-19 pandemic

**DOI:** 10.1186/s12877-023-03869-3

**Published:** 2023-04-06

**Authors:** Abeer Badawy, Mads Solberg, Aud Uhlen Obstfelder, Rigmor Einang Alnes

**Affiliations:** 1 Department of Health Sciences, Faculty of Medicine and Health Sciences, Norwegian University of Science and Technology, Larsgårdsvegen 2, Ålesund, 6009 Norway; 2 Center for Care Research, Department of Health Sciences, Faculty of Medicine and Health Sciences, Norwegian University of Science and Technology, Teknologivegen 22, Gjøvik, 2815 Norway

**Keywords:** Social communication, Older people, Technology for social contact, Pandemic, Relatives, Person-centered care

## Abstract

**Background:**

The recognition that people are social beings is fundamental for person-centered care. During the COVID-19 pandemic, the lives of older people were restricted in ways that dramatically reduced their opportunities for face-to-face contact. Limited contact with family members due to social distancing raised concerns about the well-being of older people. In Norway, interactive technologies were therefore introduced to older people to help them maintain social contact while practicing physical distancing.

**Objectives:**

This study was designed to examine how older people and their relatives experienced the use of technology-mediated communication through KOMP, a tablet-like device for supporting social contact in care facilities and homes during the pandemic.

**Methods:**

We adopted an open phenomenological approach inspired by Kvale and Brinkmann (2009) to explore how the use of KOMP became meaningful during the pandemic. The study was based on individual interviews with 4 residents in care facilities and 13 relatives.

**Results:**

The lived experiences of using KOMP among older people and their relatives revealed that adopting digital communication helped older people, and their families mitigate social distancing and maintain relationships with each other, despite the restrictions imposed by the government. Virtual involvement through KOMP afforded meaningful interconnections in the social lives of the users and their distant family members, thereby supporting their roles as parents and grandparents despite the distance, and promoting cross-generational connections among family members. Digital meetings also provided opportunities for older people and their relatives to enjoy each other’s presence in favored places, by conveying a homely atmosphere, for instance. These virtual encounters did not rely exclusively on talk as the only means of communication.

**Conclusion:**

This study suggests that communicating via KOMP was a meaningful activity for the participants. Technologies for social contact can, to some extent, facilitate person-centered care for older people in care facilities and their private homes, despite circumstances requiring social distancing.

## Introduction

Strict public measures were imposed by governments after recommendations from healthcare authorities and experts around the globe due to COVID-19, resulting in reduced face-to-face contact [1]. This dramatic decrease in social interaction and participation in communal and family activities due to physical distancing is widely considered to have had a negative impact on the mental and physical health of older people [2–6]. As a vulnerable population, older adults living at home and in care facilities were at risk of experiencing social isolation and loneliness due to restrictions on social contact [7–11]. This constituted a major societal problem, since social isolation can have negative consequences and accelerate cognitive decline [12].

In this article, we draw on the framework of person-centered care (PCC) to explore how the use of a new technology for social contact, KOMP (derived from the Norwegian *kompis*, meaning ‘buddy’), became meaningful for older people and their family members during a period of mandatory physical distancing. Respect, engagement, relationships, communication, and a focus on the individual’s values and preferences are central to PCC as a philosophy of care [13]. PCC emphasizes the importance of maintaining family ties to sustain a sense of belonging [14, 15], including relationships with friends and significant others as central aspects of both communal life and personal identity [16]. PCC is a framework that puts the whole person at the center of care, encompassing their history and family, social and cultural context, and personal strengths and weaknesses [17]. The social needs of residents in long-term care facilities, in particular, should be accommodated by helping them maintain satisfactory relationships with their families through meaningful conversation [18].

However, it is challenging for older people to maintain close familial relationships after moving to long-term care facilities due to impaired health abilities, physical relocation, and sometimes the loss of a spouse [19, 20]. High levels of social contact with family and friends have been reported to help residents adapt to living in institutional care facilities and to enhance their quality of life [14, 16, 19, 21]. When older people move to a care facility due to physical and cognitive disability, their communication patterns may change, and they become more dependent on relatives or other caregivers to determine the type and amount of social contact they have [22].

When the pandemic intensified in March 2020, and social distancing was enforced by the healthcare authorities, there was an urgent need to keep frail older adults in contact with their loved ones. Health professionals in care facilities, for instance, took the initiative to ensure that older people remained both safe and socially engaged during the crisis [10]. Communication technologies offered a wide range of possibilities for maintaining social communication despite the need to stay physically distanced at this time [23]. Computers, tablets, and cellular phones allow older people to communicate digitally, sometimes helping them overcome physical or cognitive limitations [24]. Video communication offers one possible way of maintaining or increasing communication between older people and their families [22]. It offers an alternative mode of communicating while providing an opportunity for multimodal interaction that combines verbal and nonverbal aspects of social interaction. In contrast, telephone does not allow for the same degree of nonverbal, embodied communication as video which has a greater capacity to convey information and multiple conversational cues through immediate feedback, body language, and facial expressions [22, 25].

This study contributes to our empirical understanding of communication via technologies for social contact from the perspective of older users and their relatives during the pandemic, a topic for which there is a significant knowledge gap [22, 26]. Specifically, this research adds to our knowledge about this pressing topic by investigating experiences with KOMP, a tablet-like device. This form of technology-mediated communication has been used extensively in Norwegian care facilities and homes to maintain social communication between older people and their relatives while they remain physically separated. While previous studies have investigated the perspectives of health care staff on the use of KOMP to support social contact during the pandemic and the degree to which it was normalized in caring practices [27, 28], this study specifically examines how older people and their relatives experienced the use of technology-mediated communication through KOMP for supporting social contact in care facilities and homes during the pandemic.

Approaching these accounts of KOMP usage from the perspective of PCC adds to our understanding of what role such technologies can play in the social life of older users in a time of crisis.

### KOMP

**Fig. 1:**
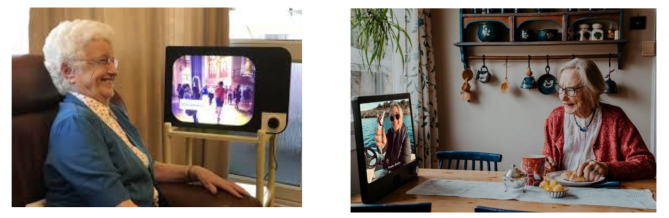
KOMP from No Isolation (©Photographer Harriet Gridley (left photo) and Estera K. Johnsrud (right photo))

KOMP, shown in Fig. 1, is an interactive tool designed to connect older people with their families by sending pictures, text messages, and video calls. KOMP looks like a small 17-inch TV screen and has built-in Wi-Fi and an eight-megapixel camera. It has a single on/off knob. It also comes with a large screen to display images (either a live video feed or rotating still images). It does not rely on a touch screen as an interface to avoid problems with capacitive sensing. It is intentionally designed with clear and loud sound to make it easier for older people to understand what others say [29].

## Methods

Inspired by Kvale and Brinkmann [30, 31], we applied an open phenomenological approach to examine participants’ experiences with KOMP and their associated meanings. As a philosophical project, phenomenology is the study of phenomena, and how everyday experiences appear to people from a first-person perspective under the assumption that there cannot be a view of phenomena from nowhere [32]. Phenomenology emerged as a deeply philosophical enterprise in the late 19th and early 20th centuries. More recently, however, phenomenology has been operationalized as an empirical and applied research program in qualitative inquiry to examine the lifeworld experiences and perspectives of individuals across various domains of life [30, 31]. This mode of inquiry seeks to describe the essence of first-person perspectives on specific social phenomena, identifying and analyzing significant units of meaning. It is achieved through a process of meaning condensation that entails reducing participants’ statements to shorter formulations and essential meanings that reproduce the immediate meanings of what has been said, usually in much fewer words than the original first-person narratives [31]. The phenomena in question (here, the experiences of communication via KOMP during a time of unprecedented social distancing) are described in an open and reflective way to reveal emergent meanings from the first-person perspective, as recalled through interviews. Such experiences may involve perceptions, thoughts, memories, imagination, and emotions [33, 34].

## Participants and recruitment

Recruitment of participants for the study began in August 2020 by contacting the manager of a short-term care facility that had recently started using KOMP. Initial contact was established via email and included a description of the study, an invitation to participate in interviews, the contact information of the first and last authors, and a consent form. The manager then forwarded the email to health care staff in the care facility. Health care professionals agreed to provide information about the recruitment process to residents and their relatives who used KOMP. Four women in the facility who used KOMP, aged between 87 and 92, were asked by the staff to participate in the interviews. Three of these residents had adequate cognitive and physical abilities for their age, and one had mild cognitive impairment. Eight close family members, including daughters, a son, a daughter-in-law, and spouses of residents in the care facility who used KOMP, also consented to requests from the staff to be interviewed for the study.

Snowball sampling was used to recruit relatives of the older people who used KOMP at home. Friends and acquaintances of the first and last authors who knew about KOMP, and had older adults in their close networks, were asked to disseminate the study description, along with an invitation to participate. Snowball sampling provided a sample of five potential respondents. The first author then sent emails to those five relatives describing the study and including a request to participate, along with the contact information of the first and last authors. Written consent to participate was obtained both in person, and digitally via email, from the five relatives.

In total, the recruitment process resulted in a sample of 17 participants (13 relatives and four residents), who shared their perspectives in 16 interviews^1^. Table 1 shows the participants, the older person’s place of residence, the older person’s age, and the interview method.

**Table 1:** Overview of the participants, their affiliations, and the interview methods

Interview	Participant	Age	Older person’s place of residence	Interview method
1	Daughter		Care facility	In-person
2	Daughter living abroad		Home	WhatsApp
3	Daughter living abroad		Home	WhatsApp
4	Grandchild		Care facility	Teams
5	Daughter		Home	Phone
6	Spouse		Care facility	In-person
7	Daughter		Care facility	Phone
8	Son and daughter-in-law		Care facility	In-person
9	Spouse		Care facility	Phone
10	Daughter		Care facility	Phone
11	Daughter		Care facility	Phone
12	Daughter		Care facility	Phone
13	Resident	92	Care facility	In-person
14	Resident	90	Care facility	In-person
15	Resident	87	Care facility	In-person
16	Resident	90	Care facility	In-person
16 interviews	17 participants			

## Data collection

Data were collected from September to November 2020. Eleven individual interviews in the care facility were conducted in November 2020. The care facility had two short-term wards where residents could stay from three weeks to more than two years. Individual interviews with four residents were conducted in person in their private rooms. Seven individual interviews were conducted with eight relatives; two were held in person at the care facility, and five were conducted digitally using the phone number of the care facility.

The five remaining individual interviews with relatives (four daughters and a grandchild) of older people living outside the care facility, either at home or in another care facility, were conducted in person on campus or digitally by phone, WhatsApp, or Microsoft Teams audio calls.

A semi-structured interview guide with open-ended questions was utilized. Examples of the questions in the resident’s interview guide include: (1) Would you talk about your reasons for using KOMP to contact your family? (2) How does the use of KOMP affect other forms of family communication (physical visitations and telephone)? (3) What are the benefits and challenges of using KOMP?, and (4) What are your thoughts about further use of KOMP? Examples of questions in the interview guide for the relative include: (1) What do you think about your relative’s social life in the care facility/home? (2) How do you experience changes in your relative’s daily life after using KOMP?, and (3) Would you describe some positive and negative experiences of using KOMP to maintain social contact?

The purpose of the interview was to determine the feelings, perceptions, beliefs, thoughts, and experiences of the participants in using digital communication to enhance social contact during the pandemic. All individual interviews were conducted by the first author and lasted between five and 37 min (average of 16 min). The interviews were digitally recorded and transcribed verbatim.

### Data management and ethics

The Regional Committees for Medical and Health Research Ethics declared that the study was outside their authority. The committee believed that the purpose of the study was not primarily to acquire new knowledge about medicine and health, but rather to investigate how residents and their relatives would experience a new technology for social contact, therefore, the study has the character of being a different type of research than medical and healthcare research. The Data Protection Official for Research at the Norwegian Centre for Research Data (NSD) – currently called Norwegian Agency for Shared Services in Education and Research (SiKt) – approved this study under reference number 108,323. At the beginning of the interviews, the authors obtained verbal consent, per the Norwegian Personal Data Act, from all participants, including residents who with cognitive impairments. Written consent to participate was also obtained from the relatives of the older persons living at home. The participants were informed that participation was entirely voluntary, and that they could withdraw from the study at any time. All data in the study were anonymized. All methods were carried out in accordance with the Helsinki Declaration.

## Analysis

The analysis was inspired by Kvale and Brinkmann [30, 31], focusing on the coding and analysis of meanings. Analysis of the interviews proceeded via five steps. First, the interview transcripts were read several times to obtain an overall sense of what they were about. We then identified the relevant meaning units (quotations) in each interview, as they were expressed by the participants, with direct significance for the investigated phenomenon (the use of KOMP to transcend social distancing). The third step was to restate the initial theme and immediate meaning of our informants’ original statements as simply and clearly as possible. We tried to read the interviews in an unbiased way to the greatest extent possible and thematize all statements from the participants’ point of view. In the fourth step, the initial theme of each meaning unit was examined considering the phenomena under scrutiny to determine the experience associated with each theme. Similar experiences were then categorized under the same subtheme. Finally, in the fifth step, homogeneous subthemes were collected under an essential theme. Three essential themes emerged from the data: (1) overcoming social distancing by adopting digital meetings, (2) staying involved in each other’s daily lives, and (3) togetherness in a digital space. Table 2 provides examples of how we condensed meanings, from units of meanings as quotations via initial themes and subthemes, to essential themes.

**Table 2:** Examples of meaning condensation from meaning units to initial themes of meanings units, subthemes, and essential themes

Interview	Meaning units (quotes)	Initial themes of meaning units	Sub-themes	Essential themes
1	“We chose to buy KOMP at the beginning of the pandemic when it was very restricted to visit my dad…it was a way to keep in regular contact with him.”	Need for social contact Differentiating between physical and digital meetings	KOMP as an alternative way to visit	Overcoming social distancing by adopting digital meetings
12	“It is useful to have KOMP when visits are limited so we can see my mom and talk to her daily.”			
10	“If I had two choices, to visit my mom or to use KOMP, I would visit her in person. But I have only one choice, to use KOMP, during the pandemic; it is good that we have an alternative.”			
10	“KOMP does not substitute for visits; however, it is an alternative way to keep in contact.”			
3	We send photos in the morning, and then we talk in the evening by video. It is pleasant.”	Maintain contact through photos and videos A pleasant way to communicate	Social contact at a distance by sharing photos and videos	Staying involved in each other’s daily lives
7	“A nice and wonderful way to get so close to my mom in the pandemic. She enjoys looking at pictures so that she will not forget her beloved family, which is very important.”			
16	“I have KOMP on all day. I like to see the photos of my family rotating all the time and enjoy seeing their birthdays.”			
15	“It is nice to have KOMP to talk with my family and to know how they are doing.”			
8	“KOMP gathers the whole family, both children, and grandchildren.”	Different family members meet in a digital room	KOMP enhances intergenerational connections	
1	“With the camera on, the video chat is more convenient for all the family members, including many siblings dispersed both across Norway and abroad.”			
3	“We sometimes have something to talk about, and sometimes we just sit or eat so mom can see us around the dining table or in different situations.”	Communicating without talking	Embodied video calls	Togetherness in a digital space
1	“Although my dad does not talk during the call, we can see him smiling, nodding or turning his head toward someone sitting in his room, such as my mom or one of us.”			
7	“My dad likes my mom to remotely join him while he is at home, or eats in the kitchen, and both of them enjoy these moments.”	Conveying a homely atmosphere via KOMP	Sharing moments in favored places	
4	“Through KOMP, I call my grandfather to show him his house or cottage”			

## Results

In the following section, we present the three essential themes that emerged from reported experiences of KOMP usage among older people and their relatives when maintaining social contact during the pandemic.

### Overcoming social distancing by adopting digital meetings

A central theme in the reported experiences was how adopting KOMP for digital meetings offered our informants a way to overcome social distancing.

#### *KOMP as an alternative way to visit*

As in many other countries, limitations on social contact were enforced in Norway to reduce the risk of infection. In mid-March 2020, care facilities placed unprecedented restrictions on physical visits to minimize the chances of viral transmission among visitors and residents. Around this time, municipal healthcare organizations such as nursing homes began facilitating KOMP use to maintain social contact for older adults. There was an urgent need to find an alternative way to help older people and their loved ones communicate safely and frequently. One daughter, in a family that had acquired the technology privately, expressed the situation as follows: “We chose to buy KOMP at the beginning of the pandemic when it was very restricted to visit my dad…it was a way to keep in regular contact with him.” Both relatives and residents emphasized how KOMP appeared to be a safe way to communicate during the pandemic when they had few alternative choices. Two other daughters, who were living abroad, appreciated that KOMP enabled them to maintain contact across a significant physical distance, and valued these digital encounters with their mother: “It is easier to keep in touch with my mom through KOMP during the pandemic. KOMP has removed restrictions of social distancing and shortened the distance through technology.”

Another daughter in our sample articulated the dilemma of the situation as follows: “If I had two choices, to visit my mom or use KOMP, I would visit her in person. But I have only one choice, to use KOMP, during the pandemic. It is good that we have an alternative.” Here, the daughter made an explicit comparison between in-person visits and digital meetings. While preferring meeting her mother in person, this was not feasible at that time, with virtual meetings presenting themselves as an adequate way of maintaining contact. The mother, who lived with multimorbidity, resided in the care facility, and required help from the staff to turn KOMP on and off. Using an iPad or smartphone was not suitable for her, due to her state of physical and cognitive decline, and KOMP was presented as a more convenient alternative. The daughter noted that even though they communicated virtually, in-person visits were not easily replaced: “KOMP does not substitute for visits; however, it is an alternative way to keep in contact.”

Several of our respondents stressed the safety aspects of KOMP, which reduced their worries about contagion when meeting physically. One relative noted how “KOMP is effective and practical when there is a risk of infection, so we talk safely with loved ones without worrying about getting infected”. Another relative, emphasized that “It is useful to have KOMP when visits are limited so we can see my mom and talk to her daily”. Most relatives and older residents reported experiences about how digital meetings with KOMP offered them a safe, practical, and effective way to communicate during the pandemic, thereby overcoming the burden of social distancing.

### Staying involved in each other’s daily lives

The second essential theme in our material revolves around how KOMP has mitigated social distancing and helped older people and their relatives to be more involved in each other’s lives despite the circumstances. By frequently maintaining social contact at a distance, KOMP promoted intergenerational connections among family members.

#### *Social contact at a distance by sharing photos and videos*

The two daughters living abroad emphasized KOMP’s photo and video functionality, enjoying these features by sending photos in the morning and talking in the evening via video link. Describing their mother’s relationship with KOMP, they made explicit comparisons with a regular TV, emphasizing that receiving video calls and photos on the device did not require their mother to have specific digital skills, thereby allowing her to maintain her independence in old age (90 years old) while living alone in her private home. In their view, KOMP enabled them to digitally care for their mother, follow up on her day-to-day issues, and spend holidays together (regardless of closed borders). They could involve her in everyday life virtually including having playtime with grandchildren, sharing meals, and having conversations.

Other participants highlighted their positive experiences and satisfaction with KOMP’s photo function too, which enabled them to conveniently share and show snapshots of things they mutually valued. These images were seen by our informants as cues for conversations, helping older people remember details about their families. One daughter described the value of KOMP during an otherwise distressing time as follows: “A nice and wonderful way to get so close to my mom in the pandemic. She enjoys looking at pictures so that she will not forget her beloved family, which is very important.” The participants in our study articulated how the photo function helped them feel closer to each other, prompting daily discussions about the meaning and significance of various old and new images, refreshing memories of their loved ones, and creating new impressions. For instance, one daughter cherished how carefully selecting specific photos from albums, such as pictures from when they were young, could spark conversations about family members.

The value of participating in family activities through KOMP was also articulated by the older users themselves. As one resident in long-term care commented to us during an interview, “It is nice to have KOMP to talk with my family and to know how they are doing.” A socially active ninety-year-old resident, who was interviewed in her room in the care facility, stressed how she was independently capable of using KOMP. Mentioning no difficulties, she was able to turn it on and off, reply to messages, make a video call to her family, and comment on the rotating photos on the KOMP screen. She received these communications from a network of 21 persons who were registered on the device’s list of contacts. The device allowed her to actively participate in her family’s social network: “I have KOMP on all day. I like to see the photos of my family rotating all the time and enjoy seeing their birthdays”. Despite their encounters being mediated by new technology, the older residents found it deeply meaningful to follow the lives of their children and grandchildren, despite these encounters being mediated virtually.

#### *KOMP enhances intergenerational connections*

KOMP also presented families with novel ways to connect across generations. One daughter emphasized how KOMP, in her experience, connected different generations of family members: “After my mom used KOMP, she said that she felt as if she had visited her grandchildren”. Family members located in different places could participate through a smartphone application, sharing text messages, photos and making video calls, thereby keeping up-to-date with information about each other. One daughter described the value of these interactions in the following words: “KOMP is a very good tool, not only for older people but for everyone in the family to be updated about what happens with each other, especially for those who live far away. KOMP gathers the whole family, both children, and grandchildren.” Similarly, the daughter of an elderly man who resided in a care facility described how KOMP had helped family members in different geographical locations across the county meet and interact digitally with her father when she was allowed to visit him in person: “With the camera on, the video chat is more convenient for all the family members, including many siblings dispersed both across Norway and abroad.” A common sentiment in these experiences of digital socialization across different generations was that of togetherness.

### Togetherness in a digital space

The participants in our study also reported on experiences with new forms of social interaction that were impossible to sustain with more conventional technologies, such as the telephone. For instance, in the virtual space that KOMP made it possible, new forms of embodied communication took place between older people and their relatives.

#### *Embodied video calls*

Video conversations allowed for a suite of nonverbal modalities were especially useful when older adults had difficulty talking. One daughter illustrated this as follows: “We sometimes have something to talk about, and sometimes we just sit or eat so mom can see us around the dining table or in different situations.” She described how their communication did not necessarily entail talking, as KOMP facilitated access to a broader range of facial expressions, gestures, and other body movements. As described by the pair of daughters who lived abroad, a meaningful interaction with their mother could sometimes be as simple as her watching them while they were eating or performing various mundane activities.

Another illustrative example of how KOMP supported nonverbal communications involved an elderly man with cognitive impairment and a speech disorder. Living in a care facility, he used KOMP to communicate with his large family. In the words of his daughter: “although my dad does not talk during the call, we can see him smiling, nodding or turning his head toward someone sitting in his room, such as my mom or one of us.” In addition, she reported how her father had begun remembering his family members by watching photos that were rotating on the device. In her view, simply watching him in his room, without any talking, helped them feel close to him.

#### *Sharing moments in favored places*

Our interviews also revealed how digital communications through KOMP afforded new forms of social contact making it possible to share special moments in favored places. In one case, a daughter described vividly how the technology helped maintain relationships between her mother, who lived in a care facility, and the father, who lived in his private home: “My dad likes my mom to remotely join him while he is at home or eats in the kitchen, and both of them enjoy these moments.” In their daughter’s words, the mother used KOMP to interact with her husband while he prepared food and ate in their kitchen at home, both appreciating a sense of place in these shared moments of togetherness, despite living in two different places.

A similar experience of sharing meaningful moments in special places via KOMP was reported by a granddaughter: “My grandfather has a better opportunity now to follow the daily life of his children, grandchildren, and great-grandchildren and be part of their lives than he did before.” She used KOMP to provide her grandfather with insights about various aspects of their daily lives, such as trips, birthdays, or family gatherings. The technology also helped her to connect him with places that were saturated with meaning for both: “Through KOMP, I call my grandfather to show him his house or cottage.” Beyond conveying a homely atmosphere, she described how KOMP made it possible for her grandfather to take an active part in her daughter’s first day of school.

## Discussion

Adopting an open phenomenological approach reveals how KOMP enabled a diverse set of meaningful experiences for older people and their relatives. Our analysis condensed these experiences into three essential themes: overcoming social distancing by adopting digital meetings, staying involved in each other’s daily lives, and togetherness in a digital space. Together, these three themes illuminate how digital communication provided new forms of social contact. In this section, we discuss these themes in light of concepts drawn from the person-centered care literature (PCC). We suggest that situating these new communication practices within the PCC framework helps us better understand how digital communication is meaningful for older people and their families.

Kitwood describes person-centeredness as a status that is granted to one person by others in the context of social relationships and in alignment with values such as respect, recognition, and trust [35]. McCormack (2004) argued, based on previous literature on person-centeredness and Kitwood’s definition, that PCC encompasses four main concepts of being: being in relation, being in a social world, being in place, and being with self [36, 37].

Following McCormack, we suggest that virtual communications via KOMP highlight new modes of being with respect to these four dimensions of person-centeredness. According to McCormack (2004), the concept of *being in relation* refers to how persons exist in relationships with other persons [36, p. 27, 37]. From a person-centered perspective, relationships are crucial for older people and include their personhood as a parent, grandparent, or spouse [36]. Being in relation can help boost the recognition and respect of older people’s role in their families’ lives.

The participants in our study overcame mandatory social distancing by adopting digital communication to maintain such social relationships. Due to a pressing need to maintain social contact between older people and their families, beginning in March 2020, several care facilities in western Norway reacted swiftly by widely adopting KOMP. Being commercially available, the device was also adopted privately by some consumers and purchased as an off-the-shelf technology, often acquired by informal caregivers on behalf of their older relatives. KOMP was also provided by some municipal home care providers. Our respondents emphasized the value of staying in contact to maintain their relationships with older family members. This is a critical issue, because the social networks of older persons typically shrink with age [38].

Using KOMP also helped older people remember their relatives through features such as photo sharing. Looking at family members and narrating about memories and places where they were born and grew up may stimulate older adults’ memory. Recalling memories not only retrieves past events but also elucidates the self through autobiographical memory, to address the question of ‘who am I?’ [39] and reflect on the past and present self [40].

*Being in a social world* is a second core feature of PCC, and revolves around how individuals need meaningful, situated interconnections in a social group [36, p. 28, 37]. Reporting their experiences to us, our informants articulated how KOMP enabled meaningful, even delightful, experiences that satisfied their desire to include older family members. Our respondents also created meaningful interconnections through conversations about pictures that were continuously shared on the screen, an experience they described as bringing them closer to each other. These were valued by the participants in our study as safe, available, and workable in an extraordinary situation. Since it is challenging to maintain regular and meaningful physical contact with older persons due to mobility issues and geographic barriers [41], having frequent and meaningful contact through KOMP can potentially support person-centeredness. In particular, the technology made it possible to communicate more frequently with distant family members, transcending closed doors and other boundaries, even transnational borders. KOMP helped them involve their distant, older relatives in meaningful social situations through video conversations and photos, including events like birthdays and other family gatherings, as well as the daily lives of children and grandchildren.

A third pillar of PCC is *being in place*; this refers to a context to which persons have their personhood attached [36, p. 29, 37]. Our informants articulated several experiences of how KOMP enabled being in place. Places connect people and provide a sense of belonging. One example of this was the wife who watched her husband prepare food and eating in their kitchen, although she was living in a care facility at the time. For this couple, the kitchen was a highly meaningful place, and the technology helped them virtually interact in this place at a distance. Interacting via KOMP helped the couple maintain relationships by conveying a homely atmosphere and connecting them to a favored place, instilling a sense of family and closeness. As such, these virtual meetings were characterized by a sentiment of togetherness, making it possible to share and recall meaningful moments. Such connections with valued places, which could be mediated via video conversations or photos, promoting health and well-being among older people.

*Being with self*, a fourth basis for person-centeredness, suggests how recognition, respect, and trust affect a person’s sense of self [36, p. 30, 37]. This concept explains how maintaining social contact with older parents or grandparents could result in recognition and respect for them. Family members showing an interest in conversing with, listening to and looking at their loved ones may support an older person’s sense of self. The example with the mother who lived in her home, and who used KOMP independently to communicate with her daughters and grandchildren, illustrates how technology can support independent living.

Our two examples of how KOMP supported nonverbal communication also highlights how technology can support a sense of self, by giving users with speech disorder the opportunity to virtually observe and participate in what the other person is doing without necessarily talking. Facial expressions, eye contact, gaze, posture, and body language were all considered expressive and meaningful acts by our informants, regardless of their virtual character, instilling a sense of being together despite significant physical boundaries between the participants. These experiences suggest that communication technologies such as KOMP have the potential to promote recognition and respect for older people as whole persons at a distance, undeterred by their health limitations.

## Strengths and limitations of the study

The data in our study were collected when there was an pressing need to maintain contact with older people during a period of enforced social distancing. Basing our analysis on data collected in a real-life context where our informants actually used this technology intensively, increases the ecological validity of our interpretations.

The sample included relatives of older people who lived in both care facilities and at home, adding a rich set of experiences to our study. Here, telephone interviews with relatives were also strength, as they helped to increase privacy, encouraging participants to be more open when discussing their personal sentiments by removing visual signals. Telephone interviews, however, are limited by a lack of visual information, which may disrupt smooth communications between the interviewer and respondent [42, 43]. For instance, a telephone interview involving an elderly spouse (86 years) to a woman who resided in the care facility ended within five minutes, because the man had a hearing disability that made the interview very challenging.

The inability to physically interview older adults who lived at home, due to pandemic restrictions, also presents a limitation. In-person interviews in homes where respondents interact with KOMP could have provided interesting perspectives on situated use of this technology.

The limited number of elderly users we were able to interview presents a limitation for our study. Restrictions during the pandemic made recruitment of more residents from additional care facilities challenging. Since health care staff facilitated our recruitment process, we were able to interview four residents in long-term care. However, due to the health situation of the residents, it was not possible to interview more residents from this particular care facility. The adequacy of our sample should be judged against the literature on sample size in qualitative research, which states that the usual sample size for phenomenological studies is between eight and twelve (with more radical views stating that a sample size as low as one person might also be adequate), depending on the study design and research questions [44, 45].

In Norwegian long-term care, the gender ratio is skewed towards more women than men (partly explained by women having a longer life expectancy) [46]. Accordingly, the number of men in the whole care facility during our data collection in November 2020 did not exceed eight men, and only one of these had access to a KOMP at the time we collected data. The main author attempted to interview this man in his private room, but due to his health condition, the interview was challenging and uninformative. Although the device was present in his room all the time, and it was occasionally used by the staff to set up meetings with family members, the resident denied that he knew or used the device. While interviewing more men could potentially have offered additional perspectives and experiences on the use of KOMP, this demographic was simply not available in the field. Future studies should therefore pay more attention to the gender dynamics of KOMP usage. However, the results presented here suggest that the question of whether an individual can benefit from KOMP is not reducible to single variables like gender but must be answered based on an individual assessment of the whole person, in line with the principles of person-centered care.

## Conclusion

An open phenomenological approach, examining experiences with a novel technology for social contact among older people and their relatives in Norway during COVID-19, revealed three essential themes: (1) overcoming social distancing by adopting digital meetings, (2) staying involved in each other’s daily lives, and (3) togetherness in a digital space. Older people and their families adopted digital communication to mitigate strict requirements for social distancing by the government. Our discussion considered these experiences in light of the framework of person-centered care. Drawing on McCormack and McCance’s concepts of *being in relation*, *being in a social world*, *being in place*, and *being with self*, our analysis shows how the respondents in our study adopted digital communications to maintain their social relationships despite significant barriers. Digital communication helped our participants create meaningful interconnections in their social world through frequent video conversations and shared photos, particularly with distant family members, thereby supporting their roles as parents and grandparents. Virtual meetings provided older people and their loved ones with a meaningful experience of being together and connected in favored places. In addition to conveying a homely atmosphere, the technology also made it possible to communicate nonverbally, thereby promoting recognition, respect, and trust for the older person despite any health disabilities. On this basis, we suggest that these technology-mediated communications have the potential for supporting PCC for older people in care facilities and at home, both during the pandemic and beyond. Future empirical and theoretical work could benefit from situating novel technologies for social contact, such as KOMP, within the framework of PCC.

## Data Availability

The datasets generated and analyzed during the current study are not publicly available to protect participants’ confidentiality but are available from the corresponding author upon reasonable request.

## Footnotes

1 One individual interviewee in the care facility had two relatives (a son and a daughter-in-law).

## List of Abbreviations

COVID-19: Coronavirus Disease 2019
NSD: Norsk Senter for Forskningsdata (Norwegian Centre for Research Data)
PCC: Person-Centered Care
